# Evaluation of the lacrimal recess of the maxillary sinus: an anatomical study

**DOI:** 10.5935/1808-8694.20130007

**Published:** 2015-10-14

**Authors:** Paulo de Lima Navarro, Almiro José Machado, Agrício Nubiato Crespo

**Affiliations:** MSc in Medical Sciences; Otorhinolaryngologist; DDS - PhD - Unicamp; Researcher - ENT Program - UNICAMP; PhD - Head of the ENT Program - UNICAMP. FCM - UNICAMP

**Keywords:** lacrimal apparatus, maxillary sinus, natural orifice endoscopic surgery, paranasal sinuses

## Abstract

The anatomical relation between the maxillary sinus and the nasolacrimal duct has gained greater importance with the advent of microsurgeries and endoscopic-assisted sinonasal procedures, and the growing use of endonasal surgery to perform middle meatus procedures and transnasal dacryocystorhinostomy. We did not find reports on maxillary sinus classification concerning its lacrimal recess, nor how often it is found.

**Objective:**

To assess how frequent the lacrimal recess can be found in the maxillary sinuses of dissected anatomical specimens.

**Method:**

We assessed 31 half-heads from cadavers. We dissected the area corresponding to the middle third of the face, by lateral access so as to be able to observe the most lateral portion of the nasolacrimal duct vis-à-vis the maxillary sinus. The maxillary sinuses were assessed by two examiners simultaneously, getting to a consensus in relation to the type of nasolacrimal duct.

**Results:**

We assessed 18 maxillary sinuses of the lateral type (58.1%) and 13 anterior sinuses (41.9%). The difference in frequency of the anterior type of maxillary type of the right side (35.7%) and left (47.1%) did not have statistical significance (*p* = 0.524).

**Conclusion:**

We found a frequency of 41.9% of lacrimal recesses in the maxillary sinuses.

## INTRODUCTION

Accurate anatomy knowledge is indispensable for any surgeon. Otorhinolaryngological surgeries involving the paranasal sinuses, especially the endoscopically-assisted ones, stress the importance of this knowledge due to the close relationship between the nasal cavity and other noble anatomical elements, such as the brain, the pituitary, the eye balls with their optical nerves and, important vascular elements, such as the internal carotid arteries, ethmoidal arteries, and cavernous sinus^1^^,^^2^. Inside the nasal cavity, the lateral wall stands out, because it holds important structures, such as the nasal conchae, the uncinate process, the infundibulum and the ethmoidal bulla. Its most anterior portion harbors other relevant structures, such as the agger nasi ethmoidal cells, the lacrimal sac and the nasolacrimal duct (NLD)^3^, ^4^, ^5^. The nasal cavity wall is also the medial border of the maxillary sinus (MS), which has the anterior recess or lacrimal recess in its anteriormost portion, called lacrimal recess because of its proximity to the lacrimal sac and the nasolacrimal duct^6^, ^7^, ^8^.

The anatomical relationship between the MS and the NLD, acquired greater importance with the development of microsurgery and endoscopic-assisted sinonasal surgeries, and by the growing use of nasal endoscopy in performing medial meatus surgery and transnasal dacryocystorhinos-tomy^9^, ^10^, ^11^, ^12^, ^13^, ^14^, ^15^, ^16^, ^17^. In medial meatus procedures, broadening the main ostium of the maxillary sinus bears the greatest risk of causing stenosis to the NLD because of their proximity. The lacrimal recess of the maxillary sinus, anterior to the NLD, thins the bone sheath that covers it, with a greater risk of being fractured during tissue resection^18^^,^^19^. In the endoscopically-assisted transnasal dacryocystorhinostomy, besides the need to master the surgical instrument required by the technique, the surgeon must consider the possibility of anatomical variations on the most anterior and medial portion of the maxillary sinus, on the lacrimal recess area, in order to properly identify the anatomical landmarks and avoid risk areas^19^, ^20^, ^21^, ^22^.

Although such anatomical variation is acknowledged, this recess is not very broadly discussed in the literature^1^^,^^2^^,^^5^. In the different literature sources investigated, we did not find reports of maxillary sinus classification as to its lacrimal recess, nor on its frequency. Therefore, the goal of the present study is to assess how frequent the maxillary sinus's lacrimal recess is identified in dissected anatomical specimens.

## METHOD

**Figure 1:**
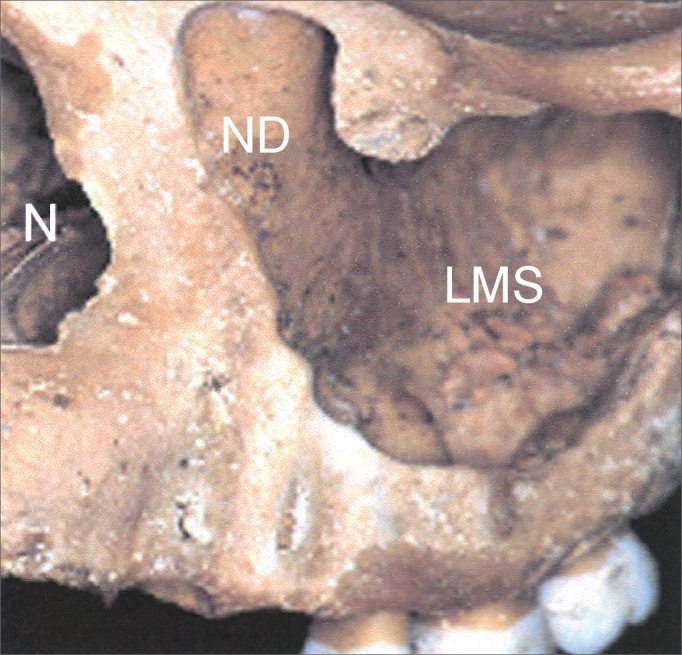
Anterolateral view of the medium portion of the face in a bony anatomical specimen. ND: impression of the nasolacrimal duct on the middle wall of the left maxillary sinus; LMS: Left Maxillary Sinus; N: Nasal pyriform opening.

In this observational, descriptive study, we assessed 31 parts of half heads from cadavers, supplied by the Morphology Department of the Dentistry School of Bauru - the University of São Paulo. The protocol for this study was approved by the Ethics in Research Committee of the School of Medical Sciences of Unicamp, under # 26/2002. The sample had anatomical specimens with good visualization of the maxillary sinus anterior wall, the medial wall and the nasolacrimal duct lateral wall. We took off the sample those specimens with signs of prior manipulation/alterations to the maxillary sinus medial wall and nasolacrimal duct. In the half-heads we dissected the area corresponding to the middle third of the face by lateral access, so as to be able to see the most lateral portion of the nasolacrimal duct vis-à-vis the maxillary sinus (Figure 1). The maxillary sinuses were assessed by two examiners at the same time, and they reached a consensus as to the type of nasolacrimal duct.

**Figure 2:**
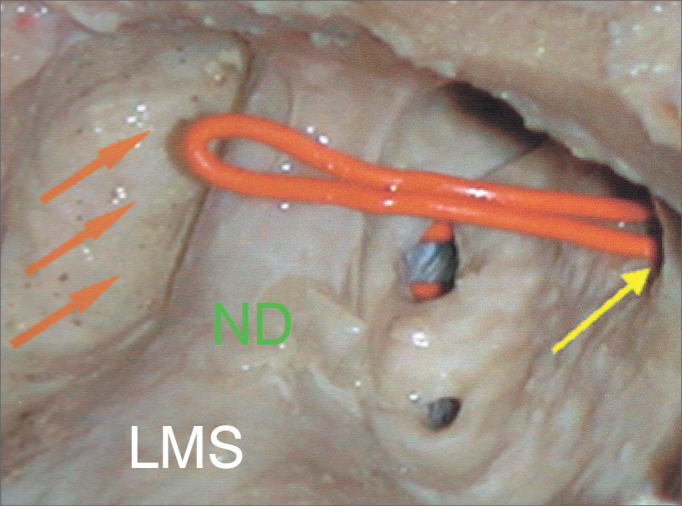
Lateral view of the dissection of the left maxillary sinus of the ANT (anterior) type. Orange arrow: lacrimal recess; Yellow arrow: main ostium of the maxillary sinus; ND: Nasolacrimal Duct; LMS: Left Maxillary Sinus.
• LAT (lateral): no depression anterior to the lateral portion of the NLD on the medial wall of the maxillary sinus, without the lacrimal recess (Figure 3).

• ANT (anterior): a depression anterior to the lateral portion of the NLD on the medial wall of the maxillary sinus, which characterizes the presence of the lacrimal recess (Figure 2);

**Figure 3:**
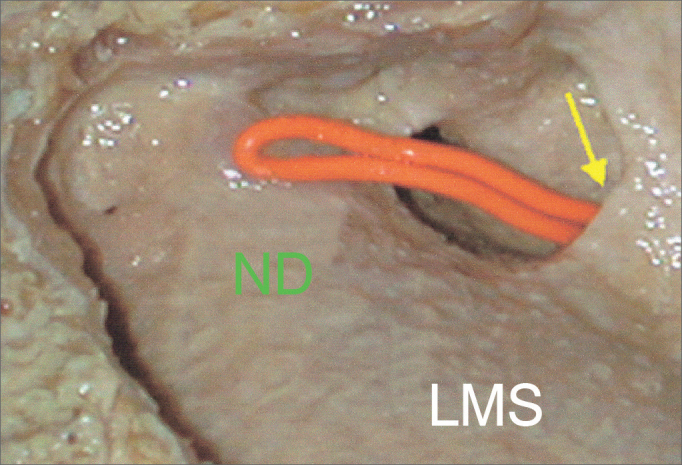
Lateral view of a dissection of the left maxillary sinus LAT (lateral) type. Yellow arrow: main ostium of the maxillary sinus; ND: Nasolacrimal Duct; LMS: Left Maxillary Sinus.

The results are presented in a table, by means of absolute frequency (n) and relative percentage (%). We used the data analysis of the Fisher's exact test at a 5% level of significance.

## RESULTS

In the anatomical specimens, we found 18 maxillary sinus of the lateral type (58.1%) and 13 of the anterior type (41.9%), in a total of 31 half-heads which were dissected (Table 1). Although there were exclusion criteria, all the samples fit the inclusion criteria, and there was no sample loss. The difference between the frequencies of maxillary sinuses of the anterior type between the right (35.7%) and left (47.1%) was not statistically significant (*p* = 0.524).

**Table 1:** Distribution of the types of maxillary sinuses observed in the dissections of anatomical specimens according to the side studied.

Dissections	LMS	RMS
	n (%)	n (%)
ANT	8 (47.1)	5 (35.7)
LAT	9 (52.9)	9 (64.3)
Total	17 (100)	14 (100)

LMS: Left Maxillary Sinus; RMS: Right Maxillary Sinus; ANT: anterior-type maxillary sinus with lacrimal recess; LAT: lateral-type maxillary sinus without lacrimal recess; n: absolute number; %: relative frequency. Fisher's exact test: *p* = 0.524.

## DISCUSSION

The frequency of the lacrimal recess of the maxillary sinus found in anatomical specimens in our study was 41.9%. Such data stresses the importance of paying attention to this variation of the normal. A careful assessment of this region before proceeding with endoscopic-assisted sinonasal surgeries helps prevent complications^1^^,^^5^, ^6^, ^7^.

Data in the literature stress this important region of the maxillary sinus, without however, inferring its preva-lence^3^^,^^8^^,^^11^^,^^17^. Our study has been a pioneer in reporting the frequency of finding the lacrimal recess in the maxillary sinus. In this study, we did not assess important structures which were previously reviewed, such as the *agger nasi*, middle concha head insertion on the lateral wall and the uncinate process. Nonetheless, due to its anatomical importance and proximity to the lacrimal recess, these structures must be targeted by future studies.

The frequency of maxillary sinus types was similar on the left and right sides, and the data obtained enabled us to say that there were no differences between them. One of the limitations of this study is the fact that we can not infer this data in terms of gender, age and ethnics, since the anatomical specimens did not have such data. The only thing we knew is that the half heads came from adults, therefore the results from this study do not apply to children and teenagers.

Other methods have been utilized as diagnostic methods to assess the anatomy of paranasal sinuses *in vivo*, such as the CT scan^17^. We suggest the use of this method to assess the frequency of having the maxillary recess in the different ethnic populations, gender and ages.

ENT is in continuous expansion and adding these procedures, which in the past belonged to other fields of medicine, brings about new challenges and the need for better understanding the nasolacrimal apparatus^13^^,^^16^. Our study tried to help in understanding the anatomy of an area associated with important and frequent procedures, such as middle meatus surgery and endonasal dacryocystorhinostomy. We also stress that the maxillary sinus's lacrimal recess is one of the most important points to be studied when one considers doing procedures in the anteriormost region of the nasal cavity and maxillary sinus, especially in interventionist procedures in the lacrimal pathways.

On dacryocystorhinostomy, a lacrimal drainage pathway is created inside the nasal cavity to reestablish drainage of the this system, when obstructed, by means of an opening more commonly carried out in the lacrimal bone. It has been historically done by ophthalmologists, by an external approach. However, with the use of endoscopy, endonasal dacryocystorhinostomy has proved to be a safe and efficacious surgical technique to treat lower lacrimal obstructions, thus becoming an alternative to treat lacrimal pathway obstruction, because of the low morbidity associated with the procedure and results matching the conventional external surgical approach^21^.

Lacrimal pathway obstruction may happen at any point of its route, and for surgery purposes they are classified into pre-sac (common canaliculus), sac or post-sac (nasolacrimal duct). The lacrimal points marks the starting of the lacrimal drainage system, followed by the inferior and superior canaliculi, which join and make the common canaliculus, extending for 1 to 2 mm, which then empties into the lacrimal sac anteriorly. Afterwards, the tear is pumped through the nasolacrimal duct, until it comes out in the nasal cavity, near the inferior meatus, at about 1.5 cm posterior to the border of the middle turbinate head.

The lacrimal sac is covered by nasal mucosa and by a bone wall (anterior by the frontal process of the maxillary sinus and posteriorly by the lacrimal bone) being located anteriorly to the unciform process. Its upper border is located above the middle concha insertion point and the lower border is near the superior portion of the inferior concha. The lacrimal sac is about 10mm in its vertical axis, of which 3-5 mm are above the insertion of the common canaliculus. Studies have shown that the lacrimal sac may extend all the way to about 8 mm above the middle concha insertion. The main valve system of the nasolacrimal duct is in its junction with the lacrimal sac and in the opening of the inferior meatus, preventing lacrimal reflux^21^^,^^22^. Thus, prior knowledge about the anatomy of the lacrimal system is paramount to understand the pathological processes involving this system considering the proper procedures for diagnosis and treatment^22^.

## CONCLUSION

By assessing the 31 half-heads dissected, we notice 41.9% of lacrimal recesses in the maxillary sinuses. There were not statistical difference in the presence of the maxillary sinus of the anterior type between the left and right sides.
